# Effect of cold ambient temperature on heat flux, skin temperature, and thermal sensation at different body parts in elite biathletes

**DOI:** 10.3389/fspor.2022.966203

**Published:** 2022-11-02

**Authors:** Thomas Blokker, Elias Bucher, Thomas Steiner, Jon Peter Wehrlin

**Affiliations:** Section for Elite Sport, Swiss Federal Institute of Sport, Magglingen, Switzerland

**Keywords:** cross-country skiing, cold stress, thermoregulation, skin temperature, heat flux, heat loss, field measurement

## Abstract

**Introduction:**

When exercising in the cold, optimizing thermoregulation is essential to maintain performance. However, no study has investigated thermal parameters with wearable-based measurements in a field setting among elite Nordic skiers. Therefore, this study aimed to assess the thermal response and sensation measured at different body parts during exercise in a cold environment in biathletes.

**Methods:**

Thirteen Swiss national team biathletes (6 females, 7 males) performed two skiing bouts in the skating technique on two consecutive days (ambient temperature: −3.74 ± 2.32 °C) at 78 ± 4% of maximal heart rate. Heat flux (HF), core (T_core_) and skin (T_skin_) temperature were measured with sensors placed on the thigh, back, anterior and lateral thorax. Thermal sensation (TS) was assessed three times for different body parts: in protective winter clothing, in a race suit before (PRE) and after exercise (POST).

**Results:**

HF demonstrated differences (*p* < 0.001) between sensor locations, with the thigh showing the highest heat loss (344 ± 37 kJ/m^2^), followed by the back (269 ± 6 kJ/m^2^), the lateral thorax (220 ± 47 kJ/m^2^), and the anterior thorax (192 ± 37 kJ/m^2^). T_core_ increased (*p* < 0.001). T_skin_ decreased for all body parts (*p* < 0.001). Thigh T_skin_ decreased more than for other body parts (*p* < 0.001). From PRE to POST, TS of the hands decreased (*p* < 0.01).

**Conclusion:**

Biathletes skiing in a race suit at moderate intensity experience significant heat loss and a large drop in T_skin_, particularly at the quadriceps muscle. To support the optimal functioning of working muscles, body-part dependent differences in the thermal response should be considered for clothing strategy and for race suit design.

## Introduction

Endurance performance is well known to depend on the external environmental conditions, particularly temperature, as well as the suitability of the clothing worn correspondingly (1–3). Indeed, a small rise or drop in core temperature (T_core_) can lead to a decrease in oxygen uptake ($\dot{V}$O_2_), aerobic power and muscle force production (4–6). While the causing mechanisms limiting aerobic exercise in the heat are complex but seem to be relatively clear (7), the ones impacting performance inherent to the cold have not received as much research attention (6). In a summary review, the latter authors have suggested possible mechanisms that impact $\dot{V}$O_2_ and aerobic performance in a cold environment. Namely changes in: temperature (lower deep body, muscle and skin temperature); metabolism (increased lactate, low glucose, fasting and increased $\dot{V}$O_2_/reduced economy); and central/peripheral circulation (reduced maximal heart rate, lower cardiac output and reduced muscle blood flow) (6). Although the individual extent to which each of these mechanisms impairs performance is not fully understood, a decrease in the different physiological temperatures seem to affect endurance exercise capacity (4, 8). Skin temperature (T_skin_) appears to be particularly important, as a larger gradient between T_core_ and T_skin_ is indicative of a higher heat loss. Despite a stable elevated T_core_, heat loss from a drop of mean T_skin_ to 27.2 °C is more than twofold compared to resting conditions (1, 9). Therefore, keeping the T_skin_ of working muscles above a critical level can pose a challenge in biathlon and cross-country skiing, where ambient temperatures can drop to −20 °C during competition.

It has been demonstrated that subzero temperatures impair endurance performance, $\dot{V}$O_2_ and exercise economy in cross-country skiers (3, 10–12). When performing at maximal intensity in a cold environment, T_skin_ decreases proportionally to the severity of the cold ambient temperature, although T_core_ increases (3). The authors, therefore, suggest that a possible responsible mechanism for the diminished performance could be the lower T_skin_, which likely induces a decrease in muscle temperature (T_muscle_). Hence, preventing a drop in T_skin_ appears relevant. Dry and evaporative heat loss may be mitigated with clothing, which provides a barrier for heat transfer between the skin and the environment (13). However, as cutaneous blood flow fluctuates across body regions, so does T_skin_ (14). Accordingly, gaining knowledge in terms of where in the body the most substantial decrease in T_skin_, and thus heat loss occurs, appears relevant to optimize clothing strategies (15). To improve skiing performance in the cold, relevant muscle groups should be appropriately covered to allow for optimal thermoregulation. However, there is a lack of research examining the thermal response to the cold of different body parts of elite biathletes in a field setting. Moreover, whether the physiological measurements diverge from the subjective thermal sensation (TS) experienced by athletes in the cold is also unknown.

Therefore, the purpose of the present brief report was to assess the thermal response of elite biathletes to a cross-country skiing bout in a cold environment in a standard racing suit. Specifically, the aim was to monitor the T_skin_, T_core_ and heat flux (HF) development in a field setting with a race suit, and identify possible differences between body parts. A second aim was to assess the subjective thermal sensation for relevant body parts over the course of the exercise session.

## Materials and methods

### Study design and participants

Thirteen national team biathletes (6 females and 7 males; age: 27 ± 4 years) performed an exercise protocol consisting of two cross-country skiing bouts on two consecutive days. HF, T_core_ and T_skin_ were measured continuously with sensors placed on different body parts, and TS was assessed before and after the bouts for different locations on the body. Anthropometrics, maximal oxygen uptake ($\dot{V}$O_2_max), aerobic and anaerobic thresholds (Table 1) were determined 7–14 days before the field tests in the laboratory of the Swiss Federal Institute of Sport Magglingen (SFISM). All participants provided informed consent to participate in this study in accordance with the internal review board of the SFISM and the Declaration of Helsinki.

**Table 1:** Anthropometric and performance characteristics.

	**Females**	**Males**
**n**	**6**	**7**
Age (y)	25.6 ± 3.9	28.2 ± 4.6
Body height (cm)	166.4 ± 7.0	180.1 ± 3.1
Body mass (kg)	62.5 ± 9.0	75.7 ± 6.5
Body fat (%)	22.5 ± 4.1	11.4 ± 1.8
$\dot{V}$O_2_max (ml·kg^−1^·min^−1^)	59.8 ± 3.7	69.7 ± 3.7
GXT AeT HR (bpm)	157 ± 8	143 ± 5
GXT AnT HR (bpm)	185 ± 6	176 ± 5

$\dot{V}$O_2_max, maximal oxygen consumption; GXT, graded exercise test; AeT, aerobic threshold; AnT, anaerobic threshold; HR, heart rate.

Mean ± SD.

### Exercise protocol

The cross-country skiing bouts (men: 4.12 km; women: 3.49 km) lasted 14.3 ± 1.3 min, and were performed in the skating technique at an intensity of 78 ± 4% of their maximal heart rate based on the information from the graded exercise test (GXT) (details below). Starting at 717 m above sea level, the ski track consisted of six uphill, six downhill and seven flat sections, crossing the low point at 698 m and the high point at 735 m above sea level. The ambient temperature and relative humidity were −1.7 ± 0.6 °C and 88.6 ± 5.3% for day 1, and −6.0 ± 0.7 °C and 97.4 ± 3.5% for day 2, respectively. The skiing bouts were performed at the same time of the day for both days. The participants' heart rate was continuously monitored with a chest heart rate monitor (HRM-Pro, Garmin, Olathe, KS, USA) and displayed on a wristwatch (Forerunner 35, Garmin, Olathe, KS, USA). The participants were instructed to adapt the skiing velocity in accordance with the exercise intensity prescription at a target heart rate corresponding to their aerobic threshold (AeT), defined in the GXT.

Athletes performed the skiing bout twice, as environmental conditions for races throughout the season change significantly and individual preferences vary greatly as to which layers are worn underneath the racing suit. As a result, athletes were explicitly instructed to pursue two clothing strategies simulating a race in warm conditions (i.e., which generally consists of underwear, socks, short base layer, race suit, headband and gloves) and cold conditions (i.e., underwear, socks, long underwear, long upper-body base layer, race suit, neck warmer, beanie and gloves), randomly assigned to the first and second days. They completed a crossover on the alternative day. The mean of the individual measurements from both days was used for further analysis. Athletes used their own skating cross-country ski equipment with standardized base wax for both days.

T_skin_ as well as HF were recorded at 1 Hz throughout the entire skiing bout, with wearable non-invasive sensors (CORE, greenTEG AG, Zurich, Switzerland) positioned directly on the skin: (I) in the middle of the vastus lateralis (thigh), (II) 2 cm above the spinous process of the T12 vertebra (back), (III) on the sternum (anterior thorax) and (IV) 20 cm under the arm pit (lateral thorax) and were held in place with a custom-made elastic Velcro-strap. T_core_ was computed with a machine-learning algorithm (greenTEG AG, Zurich, Switzerland). The calculations were derived from both the heart rate measurement as well as the HF and T_skin_ data from the lateral thorax CORE sensor. For inter-test reproducibility purposes, the exact positions of the sensors were marked with a permanent pen. In order to avoid interference with the sensor measurements placed on the back, athletes were not wearing their rifles. For each body part, the total heat loss for the entire exercise was defined as the total area under the curve from the start to the end of the skiing bout. For T_core_ and T_skin_, the reference value (START) was the average of the five values before and the five values after the start of the exercise (resulting in a 10 s average around the start time), whereas the end of the exercise (END) value was the average of the last 2 min of the skiing bout. Athletes were equipped with the sensors indoors at room temperature conditions (temperature 19.9 ± 1.0 °C, relative humidity 26.8 ± 7.8%) for a 10-min baseline measurement and to avoid premature cold exposure. Upon completion of the indoor baseline measurement, the athletes left the building and proceeded to the adjacent ski track. The subjective TS was assessed for the torso, arms, hands, legs, feet, head, neck and whole body *via* an adapted seven-point scale from −3 (cold) to 3 (hot) (16), in protective winter clothing (REF), as well as right before (PRE) and after (POST) the exercise bout in the respective race suit.

### Anthropometrics and laboratory tests

Body fat content was determined using dual-energy X-ray absorptiometry (Lunar iDXA, GE Medical Systems, Chicago, IL, USA). $\dot{V}$O_2_max was determined with the Douglas Bag technique using an uphill running test protocol to task failure on a motorized treadmill (17).

The first lactate threshold and the lactate turning point were determined during a GXT using a sport-specific treadmill protocol on rollerskis in the skating technique. At a starting velocity of 2.50 m·s^−1^ and an incline of 1, 2 or 3° based on the athlete's performance level, skiers completed recurring 5-min stages interspersed by 1 min of passive recovery. The workload was increased stepwise by a 1° incline. Heart rate was continuously measured (Firstbeat Technologies, Jyväskylä, Finland), and earlobe capillary blood samples were taken at the end of each stage to determine blood lactate concentration. The first (GXT AeT) and second (GXT AnT) lactate thresholds were calculated using the modified D_mod_ method (18). The heart rate associated with the GXT AeT was used as the target intensity during the field exercise in the cold, which corresponded to 78 ± 4% of subjects' maximal heart rate.

### Statistical analysis

Unless specified otherwise, all data are presented as mean ± SD. For the total heat losses, the areas under the HF curves were calculated following a numerical integration using Simpson's Rule (19). Normal distribution was checked with the Shapiro–Wilk test. Differences in HF were evaluated with a one-way analysis of variance (ANOVA). Changes in T_core_ were evaluated with the Wilcoxon signed-rank test. Differences in T_skin_, as well as in TS were assessed using a two-way repeated measure ANOVA (time x body part). In the case of significant main effects, pairwise *post hoc* Tukey's multiple comparisons of means were applied. The effect size was measured with partial eta squared (η_p_^2^). The significance level was set at *p* < 0.05 for all analyses. All statistics were performed using R Studio (20).

## Results

### Heat flux

**Figure 1:**
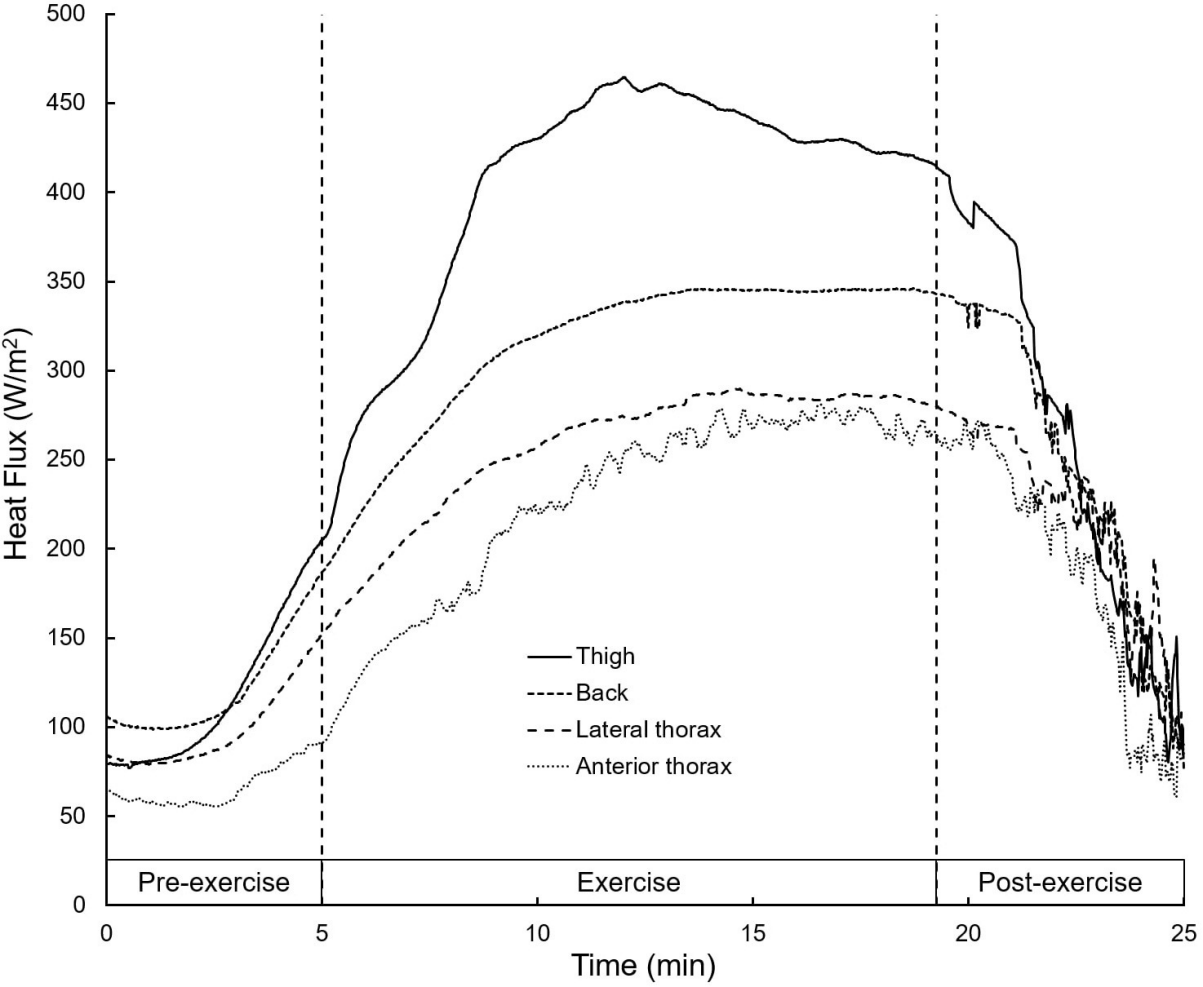
Mean heat flux measured at different body parts before, during and after exercise. Dashed lines mark the start and the end of the skiing bout.

HF increased for all body parts with the onset of exercise and rapidly decreased after the end of the skiing bout (Figure 1). Heat losses during exercise were different between body parts [*F* (3, 12) = 55.01, *p* < 0.0001, η_p_^2^ = 0.82]. Heat loss for the thigh (344 ± 37 kJ/m^2^) was higher (*p* < 0.01) than for all other body parts, while heat loss on the back (269 ± 56 kJ/m^2^) was higher than on the lateral thorax (220 ± 47 kJ/m^2^) and the anterior thorax (192 ± 37 kJ/m^2^). Lateral and anterior thorax did not differ in heat loss (*p* = 0.14).

### Core and skin temperature

**Figure 2:**
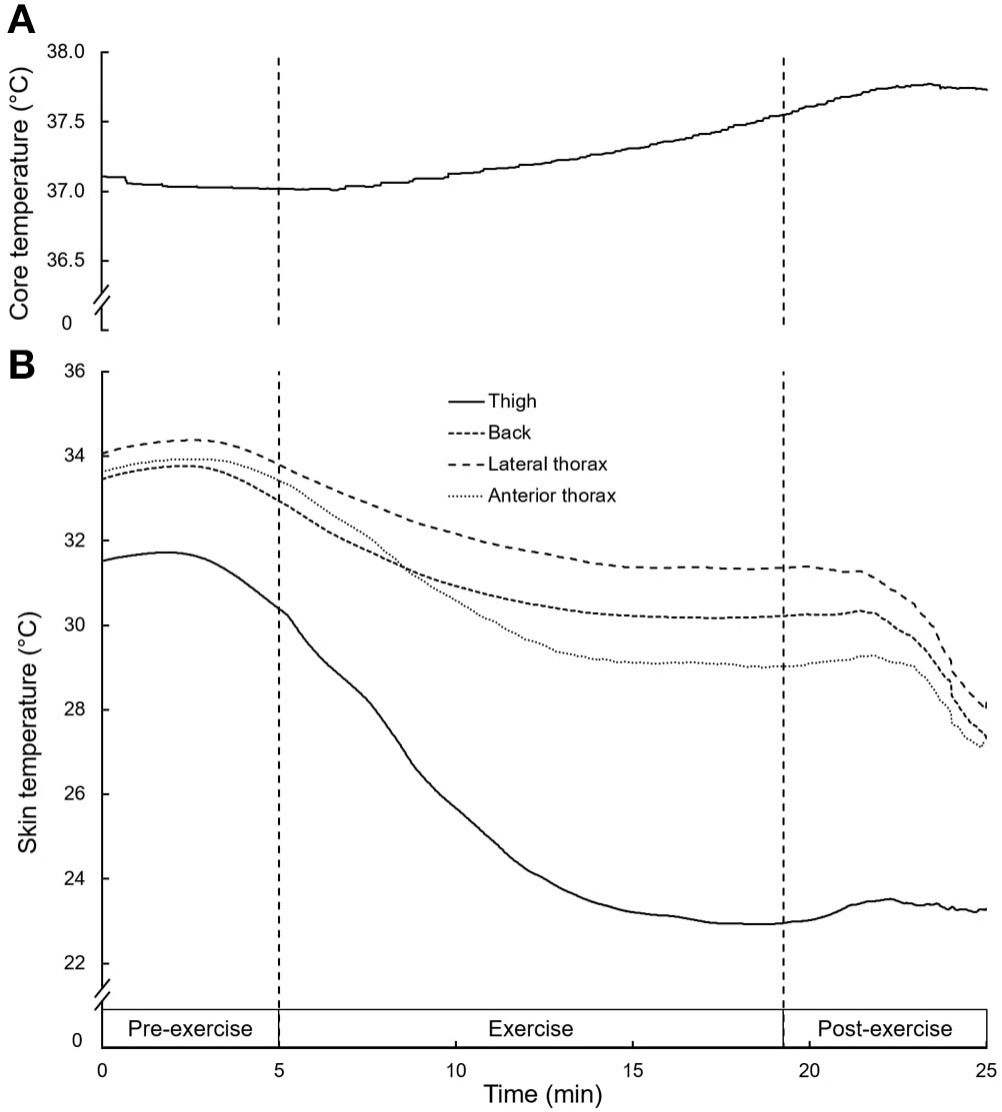
Mean core **(A)** and skin **(B)** temperature at different body parts before, during and after exercise. Dashed lines mark the start and end of the skiing bout.

From the start of the skiing bout to the end, T_core_ slightly increased from 37.0 ± 0.2 °C to 37.5 ± 0.2 °C (*p* < 0.001) (Figure 2A). T_skin_ at START were 30.4 ± 0.9 °C, 32.9 ± 0.9 °C, 33.4 ± 1.3 °C, and 33.8 ± 0.7 °C for the thigh, back, anterior and lateral thorax, respectively. Throughout the exercise, T_skin_ decreased for all body parts [*F* (1, 12) = 251.5, *p* < 0.0001, η_p_^2^ = 0.95] to 22.9 ± 1.6 °C, 30.2 ± 1.2 °C, 29.0 ± 3.2 °C, and 31.3 ± 1.6 °C, for the thigh, back, anterior and lateral thorax, respectively (Figure 2B). T_skin_ at the thigh was lower (*p* < 0.001) than other body parts at START and END, while T_skin_ for the lateral thorax was higher (*p* < 0.001) than for the anterior thorax at END.

### Subjective thermal sensation

The TS results are summarized in Table 2. The TS for the hands was the only one that dropped significantly from PRE to POST. A greater intersubject variability identifiable by larger standard deviations at PRE and POST was observed in comparison to REF.

**Table 2:** Mean thermal sensation from an analog seven-point scale according to body parts.

**Body part**	**REF**	**PRE**	**POST**
Whole body	1.03 ± 0.87	−0.15 ± 1.41***	0.12 ± 1.24***
Legs	0.88 ± 1.07	−0.26 ± 1.37***	−0.44 ± 1.40***
Hands	0.15 ± 1.26	0.51 ± 0.90	−0.53 ± 1.83^##^
Feet	0.70 ± 1.17	0.53 ± 1.36	0.06 ± 1.52*
Arms	0.81 ± 1.10	−0.72 ± 1.21***	−0.23 ± 1.16**
Chest	1.34 ± 0.75	0.27 ± 1.25***	0.50 ± 1.02***
Head	0.95 ± 0.88	0.42 ± 1.24*	−0.11 ± 1.65***
Neck	0.89 ± 0.99	0.23 ± 1.21*	0.15 ± 1.38*

Expressed in arbitrary units from −3 (cold) to +3 (warm). Mean ± SD.

^***^p < 0.001, ^**^p < 0.01, ^*^p < 0.05 indicate a significant difference from REF, ^*##*^p < 0.01 indicates a significant difference from PRE.

Neither the physiological measurements nor the TS across body parts demonstrated a significant difference between the two clothing conditions and the two test days (all *p* > 0.05).

## Discussion

The purpose of this study was to assess the thermal response of elite biathletes to a cross-country skiing bout at moderate intensity in a cold environment. This is the first study reporting HF, T_skin_ and TS measured concurrently in a field setting. The principal finding is a large decrease in T_skin_ and an increase in HF for all body parts, particularly for the quadriceps muscle, despite a moderate increase in T_core_. A second novel finding is that TS does not necessarily match the measured heat loss or T_skin_ at different body parts.

Our findings suggest that during cross-country skiing in the cold, the elevation in heat transfer that occurs is primarily driven by a large drop in T_skin_. They also corroborate analogous results conveying that T_skin_ is more dependent on ambient temperature than on T_core_ (2, 21). Presently, the number of sensors used is not enough to be aggregated into a mean skin temperature (22). However, the decrease in T_skin_ observed for every given body part in spite of a raised T_core_ is aligned with similar studies that also measured a T_skin_ drop in all measurement locations following a cross-country skiing exercise in the cold (3, 11, 23). This drop in T_skin_ seems relevant, as it has been linked to decreased cross-country skiing performance, which was suggested to be due to a lower muscle temperature (3). As it has been shown that vasoconstriction is maximal when T_skin_ is lower than 31°C (24), at which point a decline in peripheral tissue temperature occurs (25), a lower muscle temperature can be assumed. In our case, with POST T_skin_ dropping below 31°C for the thigh, back and anterior thorax, it is probable that the subcutaneous muscle tissues underneath also suffered from a drop in temperature or at least from a reduced increase in temperature in the working muscle. It is well documented that a reduced muscle temperature influences performance negatively (4, 8, 26). Moreover, it has been shown that during moderate-intensity exercise in the cold where T_skin_ is reduced, muscle temperature increases to a lesser extent than during the same exercise intensity in warmer conditions (27–29). While the exact influence of T_skin_ on muscle temperature is not entirely clear, it can be assumed that muscle temperature is influenced by the proximity of the muscle tissue to the skin surface, especially in smaller, peripheral muscle groups.

The locations exhibiting the largest decrease in T_skin_ (thigh and anterior thorax) are located on the front surface of the body exposed to an intensified effect of convective heat transfer due to the skiing velocity. It has already been shown that T_skin_ measurements on the front of the body are lower than T_skin_ measurements on the back of the body when moving at a fast velocity or against a headwind (23, 30). The severity of the decrease in T_skin_ for the thigh may be explained by the tight fit of the race suit layer on the skin, getting temporarily thinner with the flexion of the knees during the skating motion. Additionally, it can be assumed that the thigh loses more heat due to being an extremity and thus prone to a higher surface area to mass ratio.

HF appears to be strongly related to the activation and size of the underlying muscle groups and the T_skin_ at the specific body part, since muscle activation increases metabolic heat production, thereby leading to a higher temperature gradient between the musculature and the skin and hence to a higher heat loss (1). Indeed, out of the four sensor locations, the ones placed on the thigh and the back, above muscle groups majorly involved in the cross-country skiing propulsion movement, showed the largest increase in HF. Muscle heat production and T_core_ in the cold are essentially subject to exercise intensity (31) and cannot be easily regulated. Accordingly, at moderate intensity, preventing an incline in skin cooling is paramount and seems to be the most effective approach to reduce the peripheral-to-core temperature gradient and thus heat loss. As the HF increase and T_skin_ decrease were not uniform between body parts in the present study, the anterior/posterior and the upper body-lower body differences in heat loss should be taken into account in the development of ski-race suits.

Interestingly, the TS did not necessarily match the measured heat loss or T_skin_ at the different body parts. Indeed, although a drastic and significant decrease in both heat loss and T_skin_ could be measured for the legs, from PRE to POST the TS for this body part only slightly decreased from −0.26 ± 1.37 to −0.44 ± 1.40 on the −3 to +3 TS scale. Similarly, T_skin_ for the anterior thorax was significantly reduced during the cross-country skiing bout, whereas from PRE to POST, TS for that region slightly increased. Although there is a general trend toward lower values and thus a colder subjective sensation, from both REF to PRE, and from PRE to POST, only the hands were perceived as significantly colder after the skiing bout. Nevertheless, we observed a large inter-individual variation in both total heat loss and TS at PRE, and POST particularly, confirming a non-negligible wide inter-individual variability in thermal response to a set environment and work load (32). Possible explanations include body composition, sex and morphology (i.e., surface area to mass ratio) (6). The areas of the body that were perceived as the coldest at POST tended to be in the periphery, while body parts closer to the core (i.e., trunk), were sensed as warmer. This discrepancy between physiological parameters and subjective sensation is in line with other studies reporting that exercise blunts cutaneous TS in the cold (33, 34). These results may be problematic, as they attests that athletes' sensations may not accurately mirror T_skin_ and heat loss development. Therefore, athletes may not dress accordingly. As a result, biathletes and cross-country skiers may have to anticipate more physiological thermal stress than the one their subjective feelings may portray.

### Limitations

It should be considered that our results may have been affected by some limitations. First, in contrast to a climatic chamber, environmental conditions can vary substantially throughout the day and between days in the field. T_skin_ has been shown to be dependent on ambient temperature (21). Therefore, any possible variation of ambient temperature during the day may have induced inter-individual differences in T_skin_. Second, subjects could not self-select clothing based on environmental conditions and were potentially dressed either too warm or too cold. This may have influenced each subject's physiological thermal response and TS. However, this effect was mitigated by randomly allocating subjects to either type of outfit and executing a crossover the next day. On the same note, women wore thorax sensors under their sports bra, which may have induced a systematic bias by creating more insulation for these three sensors. Additionally, HF and T_skin_ for the head, which was shown to demonstrate the lowest T_skin_ value in cross-country skiing in the cold (23), was not evaluated here, as only four CORE sensors per person were available. Athletes performed the measurements at a submaximal moderate intensity, which is below the intensity they would normally ski during a race. Higher intensity exercise requires greater muscle activation; thus, generating additional heat. Therefore, different responses in HF, T_core_ and T_skin_ than measured in the present study are likely. Finally, the effect of the cold on shooting performance was not investigated. As standing passive time during shooting can vary greatly, this would have represented another uncontrollable influencing factor on the overall thermoregulatory development, which is why only the skiing part was examined.

### Practical application

This study provides novel insights into the thermal response of elite biathletes during a moderate-intensity cross-country skiing bout in the cold, with potential guidance regarding clothing strategies for skiing performance. Given our results, avoiding an excessive drop in T_skin_ is vital to prevent heat loss. Accordingly, a clothing system specifically targeting the extremities, and in particular the frontal areas where large muscle groups are involved in locomotion, seems like an appropriate strategy to minimize a decrease in T_skin_ and to minimize heat loss in temperatures well below zero. Possible concepts may include creating a thickness gradient from the front to the back, wearing more lower-body base layers, or wearing battery-based heated gloves to prevent cold hands. Keeping the thigh muscles warm before the start with appropriate insulating trousers to prevent premature cooling down of T_skin_ seems to be an additional important aspect. Further research on the thermal response in biathletes is still needed, particularly on the effect of the cold on T_muscle_ during cross-country skiing, on the effect of the shooting portion and the effect of skiing at race intensity.

## Data availability statement

The raw data supporting the conclusions of this article will be made available by the authors, without undue reservation.

## Ethics statement

Ethical review and approval was provided by the Internal Review Board of the Swiss Federal Institute of Sport Magglingen, in accordance with the institutional requirements. Written informed consent to participate in this study was provided by all subjects.

## Author contributions

EB and TS designed the study. TB, EB, and TS collected the data and performed the data analysis. TB, EB, TS, and JW interpreted the data and critically revised the paper. TB drafted the paper. All authors gave final approval for publication and agree to be held accountable for the work performed therein.

## Funding

This study was funded by Swiss Olympic and the Swiss Federal Institute of Sport Magglingen in Switzerland in collaboration with the Swiss-Ski Biathlon Federation.

## Conflict of interest

The authors declare that the research was conducted in the absence of any commercial or financial relationships that could be construed as a potential conflict of interest.

## Publisher's note

All claims expressed in this article are solely those of the authors and do not necessarily represent those of their affiliated organizations, or those of the publisher, the editors and the reviewers. Any product that may be evaluated in this article, or claim that may be made by its manufacturer, is not guaranteed or endorsed by the publisher.

## References

[1] Gonzalez RR. Biophysics of heat exchange and clothing: Applications to sports physiology. Med Exerc Nutr Health. (1995) 4:3.

[2] Galloway SD Maughan RJ. Effects of ambient temperature on the capacity to perform prolonged cycle exercise in man. Med Sci Sports Exerc. (1997) 29:1240–9. 10.1097/00005768-199709000-00018 9309637

[3] Sandsund M Saursaunet V Wiggen ØN Renberg J Færevik H Van Beekvelt MC. Effect of ambient temperature on endurance performance while wearing cross-country skiing clothing. Eur J Appl Physiol. (2012) 112:3939–47. 10.1007/s00421-012-2373-1 22426577

[4] Bergh U Ekblom B. Physical performance and peak aerobic power at different body temperatures. J Appl Physiol Respir Environ Exerc Physiol. (1979) 46:885–9. 10.1152/jappl.1979.46.5.885 468604

[5] Patton JF Vogel JA. Effects of acute cold exposure on submaximal endurance performance. Med Sci Sports Exerc. (1984) 16:494–7. 10.1249/00005768-198410000-00013 6513768

[6] Castellani JW Tipton MJ. Cold stress effects on exposure tolerance and exercise performance. Compr Physiol. (2016) 6:443–69. 10.1002/cphy.c140081 26756639

[7] Nybo L Rasmussen P Sawka MN. Performance in the heat-physiological factors of importance for hyperthermia-induced fatigue. Compr Physiol. (2014) 4:657–89. 10.1002/cphy.c130012 24715563

[8] Bergh U Ekblom B. Influence of muscle temperature on maximal muscle strength and power output in human skeletal muscles. Acta Physiol Scand. (1979) 107:33–7. 10.1111/j.1748-1716.1979.tb06439.x 525366

[9] Belding HS Russell HD Darling RC Folk GE. Analysis of factors concerned in maintaining energy balance for dressed men in extreme cold; effects of activity on the protective value and comfort of an arctic uniform. Am J Physiol. (1947) 149:223–39. 10.1152/ajplegacy.1947.149.1.223 20291966

[10] Sandsund M Sue-Chu M Helgerud J Reinertsen RE Bjermer L. Effect of cold exposure (-15 degrees C) and salbutamol treatment on physical performance in elite nonasthmatic cross-country skiers. Eur J Appl Physiol Occup Physiol. (1998) 77:297–304. 10.1007/s004210050337 9562357

[11] Wiggen ØN Waagaard SH Heidelberg CT Oksa J. Effect of cold conditions on double poling sprint performance of well-trained male cross-country skiers. J Strength Cond Res. (2013) 27:3377–83. 10.1519/JSC.0b013e3182915e7d 23539076

[12] Wiggen ØN Heidelberg CT Waagaard SH Revik H Sandbakk Ø. The effects of cold environments on double-Poling performance and economy in male cross-country skiers wearing a standard racing suit. Int J Sports Physiol Perform. (2016) 11:776–82. 10.1123/ijspp.2015-0232 26658616

[13] Havenith G. Heat balance when wearing protective clothing. Ann Occup Hyg. (1999) 43:289–96. 10.1016/S0003-4878(99)00051-4 10481628

[14] Stocks JM Taylor NA Tipton MJ Greenleaf JE. Human physiological responses to cold exposure. Aviat Space Environ Med. (2004)75:444–57. 15152898

[15] Zhang X Li J. Effects of clothing ventilative designs on thermoregulatory responses during exercise. In:Muchin VE Hu Z., editors. International Conference on Biomedical Engineering and Computer Science. (Wuhan, China: Institute of Electrical and Electronic Engineers eXpress Conference Publishing). (2010). 10.1109/ICBECS.2010.5462337

[16] Gagge AP Stolwijk J Saltin B. Comfort and thermal sensations and associated physiological responses during exercise at various ambient temperatures. Environ Res. (1969) 2:209–29. 10.1016/0013-9351(69)90037-1 5788908

[17] Sandbakk Ø Welde B Holmberg HC. Endurance training and sprint performance in elite junior cross-country skiers. J Strength Cond Res. (2011) 25:1299–305. 10.1519/JSC.0b013e3181d82d11 21081854

[18] Bishop D Jenkins DG Mackinnon LT. The relationship between plasma lactate parameters, Wpeak and 1-h cycling performance in women. Med Sci Sports Exerc. (1998) 30:1270–5. 10.1097/00005768-199808000-00014 9710868

[19] Curran J Bolstad W. Bolstad: Bolstad Functions. R Package Version 0.2-41. (2020).

[20] R Core Team. (2020). *R*: A language and environment for statistical computing. In: *R Foundation for Statistical Computing*.

[21] Saltin B Gagge AP Stolwijk JA. Muscle temperature during submaximal exercise in man. J Appl Physiol. (1968) 25:679–88. 10.1152/jappl.1968.25.6.679 5727193

[22] Ramanathan NL. A new weighting system for mean surface temperature of the human body. J Appl Physiol. (1964) 19:531–3. 10.1152/jappl.1964.19.3.531 14173555

[23] Sandsund M Wiggen ØN Færevik H Karlöf L Reinertsen RE. Thermoregulatory responses in cross-country skiers: a field study. In: Proceedings of the 12th International Conference on Environmental Ergonomics. (Boston, MA, USA). (2009).

[24] Veicsteinas A Ferretti G Rennie DW. Superficial shell insulation in resting and exercising men in cold water. J Appl Physiol Respir Environ Exerc Physiol. (1982) 52:1557–64. 10.1152/jappl.1982.52.6.1557 7107465

[25] Castellani JW Young AJ. Human physiological responses to cold exposure: Acute responses and acclimatization to prolonged exposure. Auton Neurosci. (2016) 196:63–74. 10.1016/j.autneu.2016.02.009 26924539

[26] Blomstrand E Bergh U Essén-Gustavsson B Ekblom B. Influence of low muscle temperature on muscle metabolism during intense dynamic exercise. Acta Physiol Scand. (1984) 120:229–36. 10.1111/j.1748-1716.1984.tb00128.x 6711338

[27] Claremont AD Nagle F Reddan WD Brooks GA. Comparison of metabolic, temperature, heart rate and ventilatory responses to exercise at extreme ambient temperatures (0 degrees and 35 degrees C.). Med Sci Sports. (1975) 7:150–4. 10.1249/00005768-197500720-00027 1152629

[28] Parkin JM Carey MF Zhao S Febbraio MA. Effect of ambient temperature on human skeletal muscle metabolism during fatiguing submaximal exercise. J Appl Physiol (1985) 86. (1999) 902–8. 10.1152/jappl.1999.86.3.902 10066703

[29] Meister B Collins C Mcglynn M Slivka D. Effect of local cold application during exercise on gene expression related to mitochondrial homeostasis. Appl Physiol Nutr Metab. (2021) 46:318–24. 10.1139/apnm-2020-0387 32961062 PMC8958796

[30] Mäkinen TT Gavhed D Holmér I Rintamäki H. Effects of metabolic rate on thermal responses at different air velocities in−10 degrees C. Comp Biochem Physiol A Mol Integr Physiol. (2001) 128:759–68. 10.1016/S1095-6433(01)00281-1 11282319

[31] Nimmo M. Exercise in the cold. J Sports Sci. (2004) 22:898–916. 10.1080/0264041400005883 15768724

[32] Cheung SS Ainslie PN. Advanced environmental exercise physiology. Human Kinetics. (2021). 10.1152/ajpregu.00119.2021 34283656

[33] Ouzzahra Y Havenith G Redortier B. Regional distribution of thermal sensitivity to cold at rest and during mild exercise in males. J Therm Biol. (2012) 37:517–23. 10.1016/j.jtherbio.2012.06.003

[34] Gerrett N Ouzzahra Y Redortier B Voelcker T Havenith G. Female thermal sensitivity to hot and cold during rest and exercise. Physiol Behav. (2015) 152:11–9. 10.1016/j.physbeh.2015.08.032 26343771

